# Blood transfusion is an independent predicting factor for poor outcome after cardiac surgery

**DOI:** 10.1186/cc11049

**Published:** 2012-03-20

**Authors:** J Almeida, S Zeferino, F Galas, J Fukushima, L Camara, M Lima, T Santos, M Ferreira, J Auler, R Kalil Filho, L Hajjar

**Affiliations:** 1 Heart Institute, São Paulo, Brazil

## Introduction

Blood transfusion is associated with worse outcome in critically ill patients. A restrictive strategy of blood transfusion has been advocated in patients undergoing cardiac surgery in order to avoid clinical complications related to exposure to blood components. Nevertheless, the blood transfusion rate remains elevated in clinical practice.

## Methods

We performed a retrospective study with 750 patients undergoing elective coronary arterial graft bypass (CABG) surgery, valvar surgery or combined procedure under cardiopulmonary bypass (CPB) between October 2010 and October 2011 at a university hospital cardiac surgery referral center in Brazil. We collected baseline characteristics and preoperative laboratory data, EuroSCORE, type of surgical procedure, intraoperative characteristics, blood transfusion exposure, postoperative severe complication as bleeding, low output cardiac syndrome, vasoplegia syndrome, myocardial ischemia, stroke, ventricular or supraventricular tachyarrhythmia, respiratory failure, acute renal failure, infection, ICU length of stay, hospital length of stay and mortality in 30 days.

## Results

A total of 512 patients (68.4%) was exposed to blood transfusion components. Transfused patients presented a higher number of severe clinical complications in the postoperative period compared to nontransfused patients (74 (34.1%) vs. 312 (61.9%), *P *< 0.0001). Also, the mortality rate was higher in transfused patients than nontransfused patients (1 (0.5%) vs. 18 (3.6%), *P *< 0.016). In a multivariate analysis, age, obesity, perioperative myocardial ischemia, valve disease, heart failure, blood transfusion and CPB duration are independently associated with mortality.

## Conclusion

Blood component exposure is associated with poor outcome and mortality in patients undergoing cardiac surgery. Despite the evidence that blood transfusion is associated with worse outcome, the blood transfusion rates remain unacceptably high in clinical practice.
